# Residential mobility and psychological transformation in China: From relational to institutional trust

**DOI:** 10.1002/pchj.693

**Published:** 2023-10-31

**Authors:** Yachao Wang, Shijiang Zuo, Fang Wang

**Affiliations:** ^1^ Beijing Key Laboratory of Applied Experimental Psychology, National Demonstration Center for Experimental Psychology Education (Beijing Normal University), Faculty of Psychology Beijing Normal University Beijing China; ^2^ Tiangong University Tianjin China

**Keywords:** institutional trust, relational trust, residential mobility, trust

## Abstract

As one of the important drivers of social change in China, residential mobility has caused a dramatic change in the interpersonal environment, but it remained little known how residential mobility would influence the basis of interpersonal interaction—trust. The present research aimed to explore the effect of residential mobility on two kinds of trust, relational trust and institutional trust, by two studies. Study 1 explored the correlational relationship between regional residential mobility and two kinds of trust using data from the China General Social Survey 2010 and the Sixth National Population Census of China, and analyzed the data using hierarchical linear modeling. Study 2 switched to the individual level and investigated the causal relationship between individual residential mobility and two kinds of trust in the laboratory using the writing task for priming residential mobility and the situational selection task for trust. Study 1 found that individuals exhibited lower relational trust when they lived in a region of higher residential mobility. For institutional trust, the indicator about the permission to register household in inflow cities could significantly positively predict this. Study 2 found that the primed mindset of high (vs. low) residential mobility reduces relational trust and enhances institutional trust. In conclusion, the present research revealed that residential mobility promotes the transformation of individuals' trust mode from relational to institutional trust in social life, thus expanding the research field of residential mobility as a socioecological factor and extended the understanding of psychological transformation under the background of social change in China.

## INTRODUCTION

Population migration has now become a major theme of this rapidly changing era globally. In recent decades, large numbers of migrant people in China continue to converge in economically developed and metropolitan areas, along with the boom of economic development of inflow cities. According to the data released in the Seventh National Population Census of China in May 2021, the number of China's floating population was close to 376 million in 2020, accounting for 26.62% of the total population, which was equivalent to approximately one‐fourth of the population being in a migratory state. Compared to the data from the Sixth Census, the number of China's floating population had increased by around 155 million, with a growth rate of 69.73%. These figures indicated a sharp increase in the scale of China's floating population, signaling the arrival of the “era of migration” in China (Zhou, 2021). What is even more astonishing is that during the Third Census of China in 1982, the proportion of the floating population to the total population was only 0.7% (Cheng & Duan, 2021). In other words, at that time, only seven out of every thousand Chinese people were floating. By 2020, it had already become one out of every four. Over the course of just 40 years of reform and opening up, it seems that China's population has been experiencing a profound transformation from “rural China” with low mobility to “migratory China” with high mobility (Cheng & Duan, 2021; Duan et al., 2019). These changes can be regarded as a very specific manifestation of social change in China in terms of population mobility. As an unignorably social phenomenon, the massively floating population may bring about great impacts on the interpersonal environment as well as individuals' inner world. Residential mobility, a key indicator of population migration, has been found through research to play an important role in many psychological outcomes, such as emotions, self‐construal, group identity, and interpersonal relationships (Choi & Oishi, 2020). In the present study, we are interested in whether trust, as the basis of interpersonal interaction, will vary along the change of interpersonal environment induced by residential mobility in China.

### Residential mobility and interactive strategies with interpersonal environment

Residential mobility can be defined as the degree to which people in a given area change residence over a given period of time (Oishi, 2014). At the individual level, residential mobility is conceptualized as the number of residential moves one person experienced during a certain period or expects to implement in the future. At the macro level, it is conceptualized as the proportion of residents in a given neighborhood, city, state, or country who moved during a certain period or expect to move in the future (Oishi, 2010). In previous research, residential mobility is regarded as a typical socioecological environmental factor, specifically, an interpersonal environmental factor, shaping individuals' interaction strategies with the interpersonal environment in a relatively distant and objective way (Oishi, 2014). For example, frequent movers tend to hold conditional group identification (Oishi et al., 2009), establish a broad and shallow social network (Oishi & Kesebir, 2012), and their subjective well‐being is based more on self‐esteem than social support (Oishi, 2010). As an essential basis of interpersonal interaction, trust may be affected by it too.

### Relational and institutional trust

Trust is a psychological state comprising the intention to accept vulnerability based on positive expectations of intentions or behaviors of another (Rousseau et al., 1998). It works as the premise for establishing and maintaining interpersonal relationships and the basis for social interaction (Davis et al., 2000). Moreover, it is the cornerstone for promoting economic development and the guarantee for facilitating the normal and orderly operation of social interactions (Knack & Keefer, 1997).

Under the background of economic development and the continuous growth of the floating population, however, trust within Chinese society has been experiencing worrying changes. In a survey of residents in seven cities in 2011, 1943 respondents scored an average of 59.7, that is, a failing grade, on social trust (Rao et al., 2013). Other surveys also showed that the overall social trust of Chinese people had declined sharply in recent years (Gao, 2016b; Zhang & Xin, 2019), indicating that Chinese society has been going through a serious “trust crisis” (Jing, 2013).

Nevertheless, it does not mean that there is only a downward trend in Chinese trust, because the degree of individuals' trust towards different objects varies a lot in China. It was found in surveys that both urban and rural residents trusted their family members, blood relatives, and close friends the most, followed by colleagues, leaders, neighbors and friends who interact with them frequently, and the most distrusted were random strangers such as netizens, manufacturers, and so on (Hu & Li, 2006; W. M. Li & Liang, 2002; Tong, 2006). This kind of trust mode obviously reflects the unique relationship pattern of stratified closeness and hierarchical order among Chinese people (Tong, 2006), which was formed in traditional Chinese society and was recognized by both Chinese and foreign scholars (called “ChaXu GeJu” in Chinese; Fei, 1985), highlighting the importance of the network of ones' intimate society on trust. Furthermore, Chinese society is gradually moving from tradition to modernity during the process of social change (Zhu, 2011), so researchers have noticed that in addition to interpersonal trust, a new kind of trust, which is based on contracts, systems, and institutions, has been emerging quietly. As was shown in the survey by Rao et al. (2013), Chinese urban respondents showed the highest trust towards government agencies (69.2), close to the level of “basic trust”, while the public's trust towards commercial industries was the lowest (51.8), falling into the level of “basic distrust”. Nonetheless, there is quite different levels of public trust in different types of government agencies. For example, the public's trust towards hospitals was only 53 points (basic distrust), but trust towards the central government was up to 76.8 points (high level of basic trust), which reflects the complex and contradictory nature of this kind of trust in China.

Since the network of intimate society exerts an important influence on Chinese individuals' trust and trust towards people is quite different from trust towards institutions, it is not appropriate to adopt the concepts and measurements of trust used in Western research directly. For example, some widely used trust measurements (such as the Rotter Interpersonal Trust Scale) usually mix trust among people and institutions. The present research referred to the theory of five prototypes of trust in Chinese culture (Wang et al., 2006) and focused on relational and institutional trust. Specifically, relational trust is defined as individuals' trust towards specific people who interact with them inside the network of their intimate society, and institutional trust is defined as individuals' trust towards institutions and nonspecific people who interact with them under the operation of institutions. They are different in objects, forming foundations, and so on.

The question is whether these two kinds of trust—relational trust and institutional trust—would be affected by the growth of residential mobility in China. Specifically, it is worth studying how relational trust will change when the original interpersonal pattern suffers a strong impact due to residential mobility, and how institutional trust will develop when residential mobility is increasing, especially when the original trust among people no longer helps to cope with the uncertainties of social life. In short, the current study aims to explore potential effects of residential mobility on two kinds of trust in China.

### The relationship between residential mobility and relational trust

Relational trust has some important foundations, including close emotional connections, repeated interaction experiences, and an effective reputation‐monitoring mechanism. The traditional society of China was an agricultural society based on a subsistence farming economy (Tao & Wang, 2006), namely, “*Gemeinschaft*” (a residentially stable and traditional community; Oishi, 2010), in which people lived together in family or clan units with little or no mobility throughout their lifetime. In such a society, relationships between people were extremely stable, mostly determined by blood or geopolitical factors, with a strong sense of predestination. People were completely familiar with each other and had deep emotional connections. In such a social network, interpersonal interaction was equivalent to playing infinite times repeated games with limited people. To ensure the maximization of long‐term interest, both participants in the interaction must abide by the trust principle, which is supported indirectly by evidence that residents showed strong kin‐based cooperation in Yasawa Island where geographic factors limited residential mobility (McNamara & Henrich, 2017). If someone violated the trust principle, they would be sanctioned by the reputation‐monitoring mechanism (Rand & Nowak, 2013), at least bearing the risk of being punished and disgraced.

However, residential mobility changes this completely. In a society where people can freely move around and choose their habitats, acquaintances become remote interactive objects for they are always thousands of miles away and may not be able to provide timely support when needed (Oishi & Talhelm, 2012), and hence emotional connections are greatly weakened. Instead, abundant interactions with strangers, which are always one‐off, anonymous, and loose, constitute the greatest part of one's social life (Schug et al., 2010), reducing opportunities for long‐term repeated interactions. Moreover, high residential mobility makes it hard for people to gather information about a person and hinders reputational information transmission (Zuo et al., 2018). Therefore, one's actions in the last game can be completely unknown in the next round and someone's transgression before will not be identified or sanctioned by group pressure or conscience any more (Zuo et al., 2022). As a result, the binding effect of reputation within the network of acquaintances will disappear gradually.

Therefore, we proposed Hypothesis 1:

Hypothesis 1 Residential mobility reduces relational trust.

### The relationship between residential mobility and institutional trust

In contrast, the foundations of institutional trust are wide interaction with nonspecific individuals and the binding effect of institutions. Residential mobility provides fertile ground for the formation and consolidation of these foundations.

At first, residential mobility provides new relationship resources, that is, one‐off interactions with a large number of strangers. It was found that frequent movers could develop a new set of strategies for maintaining relationships with strangers, such as the expansion of the social network (Oishi et al., 2013), interaction in a broad and shallow way (Oishi & Kesebir, 2012), and the friendship compartmentalization strategy (Lun, Roth, et al., 2012). Compared with stable residents, mobile residents are more willing to help outgroup members (W. Li et al., 2019). In a mobile state, the human nature of socializing could manifest as their attempt to get along with, cooperate with, and depend on continuous new relationships, which might encourage them to trust nonspecific objects tentatively in everyday life.

Next, mobile residents also need some new guarantee mechanism to regulate their interactions with strangers. On the one hand, interaction with strangers is full of uncertainty. A recent study found that residential mobility decreased trust‐building intention towards strangers (Zhao et al., 2021), because of the potential risk of the interaction with strangers in a mobile environment. Compared to untrustworthy strangers, specific institutions are obviously much more stable and reliable; moreover, they can also play a role in monitoring and regulating the interaction of strangers. On the other hand, organizations and institutions can act as the new guarantee mechanism. It was shown that frequent movers preferred egalitarian helpers rather than loyal helpers (Lun, Oishi, & Tenney, 2012). Organizations and institutions are the “egalitarian helpers” for common people. Meanwhile, frequent movers are familiarity‐seeking and familiarity‐liking, and they tend to go shopping in national chain stores rather than local stores (Oishi et al., 2012). Institutions and organizations provide the relatively familiar and stable existence for people in society to some extent. Therefore, frequent movers would be more willing to trust stable organizations and certain systems to protect themselves in social life, in other words, institutional trust could be a kind of guarantee for the normal functioning of the mobile stranger society.

We proposed Hypothesis 2:

Hypothesis 2 Residential mobility enhances institutional trust.

### The present research

Two studies combing regional and individual levels were designed to test our hypotheses. Study 1 explored the relationship between regional residential mobility and two kinds of trust using data from social survey and population census. Study 2 investigated the causal relationship between individual residential mobility and two kinds of trust in the laboratory.

## STUDY 1: THE RELATIONSHIP BETWEEN REGIONAL RESIDENTIAL MOBILITY AND TWO KINDS OF TRUST

Study 1 explored the relationship between regional residential mobility and the average degree of trust among residents in China, with provinces serving as the unit of analysis. It was predicted that, as residential mobility increased at the regional level, relational trust would decrease, and institutional trust would increase.

### Method

#### Measures

##### Regional residential mobility

**FIGURE 1:**
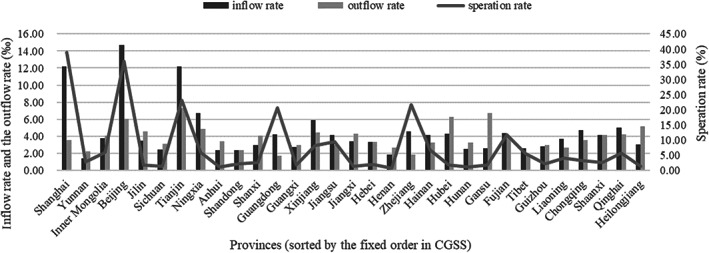
The inflow rate, outflow rate and separation rate of different provinces in Study 1.

The overall population mobility in a specific period and area, counted by the frequency of moving over a wide range (e.g., moving across provinces) in general, was chosen as the indicator (Oishi, 2010). First, to match the collection year of data for regional residential mobility, the data on trust were selected for 2010. Then, since the data source for mobility included data for both inflow and outflow directions (i.e., moving into or out of a province), directions of residential mobility at the regional level were distinguished and taken into consideration together, for they might exert different effects on interpersonal environment. Specifically, two different indicators were used to represent the degree of population inflow at the regional level. One is the percentage of people whose provinces of household registration location are different from provinces where they currently reside on the overall permanent residential population, called the separation rate, calculated from the China Sixth National Population Census in 2010 which could be obtained from the official website of the National Bureau of Statistics in China (NBSC; http://www.stats.gov.cn). The other one is the percentage of people who migrated their household registration into a new province in a given year on the overall amount of household registered population in this new province, called the inflow rate, calculated from the Population Statistics of Counties and Cities in China which was recorded by the Ministry of Public Security. The critical difference between them is whether people received permission to register their new household in inflow cities, which is extremely meaningful for studies about residential mobility in China because of the unique household registration system. For population outflow, the percentage of people who migrated their household registrations out of a province in a given year on the overall amount of the household registered population in this original province was used because the outflow of population could only be counted using official statistics based on the household registration system. It was called the outflow rate, similar to the inflow rate. The data for the above three indicators is presented in Figure 1. The format of Figure 1 follows Paulson et al. (2021) and Hafeez et al. (2023). Moreover, some regional data were collected to serve as control variables, such as per capita gross domestic product (GDP), land area, and permanent residential population of each province.

##### Relational trust and institutional trust

Data for trust were obtained from the Chinese General Social Survey 2010 (CGSS 2010; http://cgss.ruc.edu.cn), which was conducted by the National Survey Research Center in Renmin University of China. This large social survey recruited 11,783 adult residents as interviewees by a multi‐hierarchical stratified sampling, covering 31 first‐class administrative divisions in mainland China. Two questions in the survey, D2 and D3, asked interviewees their degree of trust in different kinds of people (e.g., family members, friends) or in different institutions (e.g., central government, local media) on a five‐point scale (1 = *totally distrust* to 5 = *totally trust*). After deleting some ambiguous items, the average score of the former was counted as the indicator of relational trust (*α* = .79), and the average score of the latter was counted as the indicator of institutional trust (*α* = .89). When the score was higher, the level of trust was higher. After removing missing values, 10,507 interviewees were included in the analysis (5359 female, 5148 male; 62.5% urban residents, 32.5% rural residents), with an age range 17–94 (*M* = 46.80, SD = 15.55). The distribution of participants across provinces is presented in Table 1.

**TABLE 1:** The distribution of participants across provinces in Study 1.

Province	*n*	Province	*n*	Province	*n*
Tibet	19	Shanxi	285	Hunan	452
Hainan	95	Yunnan	307	Shanghai	458
Ningxia	96	Shaanxi	335	Jilin	473
Qinghai	99	Tianjin	354	Guangdong	477
Inner Mongolia	100	Guangxi	354	Beijing	499
Xinjiang	100	Anhui	383	Zhejiang	538
Gansu	200	Liaoning	395	Sichuan	550
Guizhou	257	Shandong	412	Hubei	570
Hebei	261	Jiangxi	436	Heilongjiang	588
Fujian	264	Jiangsu	438		
Chongqing	265	Henan	447	Total	10,507

### Results

The indicators of residential mobility were associated with each interviewee by the same code for each province. Hierarchical linear modeling (HLM; Bryk & Raudenbush, 1992) was used to test whether regional residential mobility was related to the degree of trust among residents. Two models were established where two kinds of trust served as dependent variables. The models were used to analyze the effect size of residential mobility (regional variables: separation, inflow, and outflow rates) and other variables (individual variables: gender, age, and socioeconomic status; regional variables: per capita GDP, land area, and permanent residential population of each province) on trust (see Table 2). Results of the intraclass correlation (ICC; Kreft & De Leeuw, 1998) revealed that 5.2% of the variability in relational trust (*χ* ^2^(30) = 488.49, *p* < .001) and 9.2% of variability in institutional trust (*χ* ^2^(30) = 883.35, *p* < .001) were attributable to the regional‐level variables, providing an empirical basis for testing the effect of regional residential mobility on trust. The multilevel model was specified as follows:

**TABLE 2:** Hierarchical linear model analysis of two kinds of trust in Study 1.

Model (based on different dependent variables)	*χ* ^2^ in zero model (*df* = 30)	Coefficients of predictive variables on the individual level in random‐coefficients regression model (*df* = 30)	Coefficients of predictive variables on the regional level in intercepts‐ and slopes‐as‐outcomes model (*df* = 24)
Relational trust	488.49***	Gender (*γ* = .041, *p* = .001)	Separation rate (*γ* = −.014, *p* = .073)
		Age (*γ* = .002, *p* < .001)	Inflow rate (*γ* = .026, *p* = .128)
		SES (*γ* = .023, *p* < .001)	Outflow rate (*γ* = −.007, *p* = .761)
			Per capital GDP (*γ* = .003, *p* = .455)
			Land area (*γ* = .002, *p* = .018)
			Permanent resident population (*γ* = −.001, *p* = .445)
Institutional trust	883.35***	Gender (*γ* = −.005, *p* = .728)	Separation rate (*γ* = −.012, *p* = .116)
		Age (*γ* = .006, *p* < .001)	Inflow rate (*γ* = .074, *p* = .021)
		SES (*γ* = .025, *p* < .001)	Outflow rate (*γ* = −.041, *p* = .232)
			Per capital GDP (*γ* = −.008, *p* = .053)
			Land area (*γ* = .001, *p* = .337)
			Permanent resident population (*γ* = −.001, *p* = .970)

*Note*: To test the hypotheses step by step, we conducted zero model, random‐coefficients regression model and intercepts‐ and slopes‐as‐outcomes model, following the methodology of hierarchical linear modeling. Some important results are shown in the table above. Gender is dummy‐coded as 0 for females and 1 for males. Variables are added grand centered except gender.

*** *p* < .001.

Level 1 (individual level):

$$\text{Degree of trust}=\beta_{0}+\beta_{1}\;\left(\text{gender}\right)+\beta_{2}\;\left(\mathrm{age}\right)+\beta_{3}\;\left(\mathrm{SES}\right)+r.$$

Level 2 (regional level):

$$\beta_{0}=\gamma_{00}+\gamma_{01}\;\left(\text{separation rate}\right)+\gamma_{02}\;\left(\text{inflow rate}\right)+\gamma_{03}\;\left(\text{outflow rate}\right)+\gamma_{04}\;\left(\mathrm{per}\;\text{capital}\;\mathrm{GDP}\right)+\gamma_{05}\;\left(\text{land area}\right)+\gamma_{06}\;\left(\text{permanent residential population}\right)+u_{0}.$$

$$\begin{array}{c} \beta_{1}=\gamma_{10}+u_{1}\\ \beta_{2}=\gamma_{20}+u_{2}\\ \beta_{3}=\gamma_{30}+u_{3} \end{array}$$

Coefficients *γ*_10−_ *γ*_30_ represent the slope of gender, age, and SES, respectively, on predicting trust. Coefficients *γ*_01−_ *γ*_06_ represent the influence of the separation rate, inflow rate, outflow rate, per capital GDP, land area, and permanent residential population of each province on the intercept of the degree of trust.

The HLM analysis revealed that for relational trust, the separation rate could marginally, significantly, and negatively predict the degree of relational trust among residents (*γ* = −.014, *p* = .073), while the inflow rate was not significantly correlated with it (*γ* = .026, *p* = .128). For institutional trust, the inflow rate could significantly and positively predict the degree of institutional trust among residents (*γ* = .074, *p* = .021). However, the separation rate could not significantly predict it (*γ* = −.012, *p* = .116). Finally, the outflow rate was not significantly correlated with the two kinds of trust (*p*s > .232).

### Discussion

Study 1 examined the relationship between regional residential mobility and two kinds of trust by combining the regional and the individual level of data, revealing many meaningful results.

First, compared with population outflow, population inflow has a greater influence on trust at the regional level. Considering that residential mobility affects the interpersonal environment, the impact of the influx of large numbers of strangers will be more prominent than the outflow from specific regions. Therefore, the relatively small effect contributed by the outflow rate was not surprising.

Additionally, relationships between two indicators in the direction of inflow and relational trust were subtly different. It was consistent with the hypothesis that the separation rate could negatively predict relational trust, although the effect is marginally significant. Considering the limited sample size of 31 at the regional level, this result might be acceptable. However, the inflow rate was unrelated to it. This is probably because the separation rate not only represented the residential mobility in this region for some time but also implied possible mobility in the future. People who moved into a new province without permission to register their households would be more likely to move to a new place again in China. Conversely, if they were permitted to register new households, they would have a greater possibility to settle there and interact with indigenous inhabitants repeatedly, and the anticipated interpersonal environment would be more stable for them in the future. Thus, only the relationship between the separation rate and relational trust showed the negative link.

Furthermore, results for institutional trust were more surprising and thought‐provoking. While the inflow rate could significantly positively predict institutional trust, the separation rate could not significantly predict it. This could be explained by social reality about the critical difference between these two indicators to some extent. On the one hand, under the unique household registration institution, the inflow rate might act as a mirror of the flexibility and openness of local social systems, especially systems related to household registration. This situation implies that when people can choose freely where to reside and experience benefits of social systems, that is, institutions are good enough, institutional trust in that local area would increase, which successfully verified our hypothesis. On the other hand, the separation rate reflects the social situation that a large number of immigrant population cannot register permanent households in inflow areas. Those floating people could enjoy benefits of social systems about choosing jobs and residence freely as well as convenience of modern life, but had to confront various institutional barriers after mobility, such as household registration restrictions on their children's college entrance examination. The former would lead to a rise in institutional trust and the latter would lead to a decline, so those different tendencies would ultimately cancel each other out, leading to insignificant results. In other words, these results implied that different indications of inflow direction might reflect different degree of soundness of local institutions, which might exert different impact on institutional trust, so it was necessary for the present study to distinguish indicators of inflow direction and consider the impact of household registration.

In summary, the results in Study 1 implied the complicated effects of residential mobility on trust in China by a multilevel approach. The following study switches to the individual level and further explores the causal relationship between residential mobility and two kinds of trust. Furthermore, Study 1 revealed that the results for institutional trust might be more easily influenced by social reality, objectively or subjectively. Therefore, when investigating the causal relationship between residential mobility and two kinds of trust in Study 2, effective methods are used to control the possible influence of social reality.

## STUDY 2: THE CAUSAL RELATIONSHIP BETWEEN INDIVIDUAL RESIDENTIAL MOBILITY AND TWO KINDS OF TRUST

Study 2 aimed to examine the causal relationship between individual residential mobility and two kinds of trust using a laboratory experiment. To eliminate the potential effects of realistic social factors and determine the theoretical relationship between variables, the experiment was conducted under a fictitious social background that was set to provide clear and effective institutions for people. Therefore, Study 2 did not have to consider the complex relationship between residential mobility and institutional trust like in Study 1, nor did it have to distinguish the direction of mobility.

### Method

#### Participants and procedure

The sample size was determined by G*Power in advance (Faul et al., 2009). The effect size in previous studies was at the average level (Oishi et al., 2012; Zuo et al., 2018), so we set the variables to *α* = .05, power (1‐*β*) = 80%, and *d* = 0.50. This showed that 102 participants were needed. Then 107 college students were recruited to participate in the experiment by network platform. They were allocated randomly into the experimental (*n* = 58) and control (*n* = 49) groups. However, nine participants in each group failed to pass the manipulation check and their data were excluded. Finally, there were 89 valid participants (57 female, 32 male), 49 in the experimental group (31 female, 18 male) and 40 in the control group (26 female, 14 male). The age range was 18–28 (*M* = 21.69, SD = 2.50).

After random allocation into two groups, participants were asked to read instructions about a fictitious social background. Then, they completed a written task for priming the mindset of residential mobility and manipulation check items. Finally, they finished the situational selection task of trust and filled in some demographic variables. It took 15–20 min to complete the whole experiment. Participants received 20 Yuan as reward. All procedures involving human participants in this study were approved by the Ethics Review Committee of the Faculty of Psychology, Beijing Normal University, and with the 1964 Helsinki Declaration and its later amendments or comparable ethical standards. Informed consent was obtained from all participants included in this study.

### Materials

#### The fictitious social background

The instructions were as follows:

“Imagine when you wake up one day, you start a new life in a fictitious society called Yooomhaa, where the economy is developed, social institutions are perfect, and daily life is convenient. In other words, Yooomhaa is an ideal livable world. You have lived here for many years, and now you are about to graduate from university, having found a job…”

These instructions followed a similar method used in previous research for setting the social background (Sánchez‐Rodríguez et al., 2017). To guarantee the continuity of the social background, participants were reminded that they were still living in Yooomhaa during the process of the experiment.

##### Residential mobility

Participants were primed with the mindset of high residential mobility by a typical written task (Oishi et al., 2012). Participants in the experimental group (mobile condition) were required to imagine the following scenario: “*You got a job that you have always wanted. However, this job involves moving to a different city every other year in the following 10 years*”. Conversely, participants in the control group (stable condition) were required to imagine the following scenario: “*You got a job that you have always wanted. However, this job involves living in the same city every year in the following 10 years*”. Then, all participants were required to answer three questions about their life experience in the space provided (Zuo et al., 2018): (1) “*what would it be like to have such a lifestyle*”, (2) “*what would be the pros and cons about this kind of life*”, (3) “*how would this state of life influence your relationships with other people, acquaintances or strangers*”. The text of each answer was required to be at least 50 Chinese characters to guarantee the effect of priming. Then participants answered true or false on four items used as manipulation check, for example, “*In the following ten years, I need to move frequently in Yooomhaa*”, or “*For some time in the future, I will almost never change my residence in Yooomhaa*”. If they filled in wrong answers, such as choosing “false” to both items above, their data were excluded.

##### Trust

For ordinary people, trusting a relationship or an institution, namely, by relying on acquaintances or strangers, is always an either‐or choice made case by case in daily life. Hence, 10 real‐life contexts covering important and common life events were designed, including hunting for jobs, buying cars, choosing schools for children, having an operation, and so on (see Appendices). Participants had to make a forced choice between opposing selections under each context: trusting close relationships or trusting social institutions. The overall preference across all contexts was regarded as one's trust preference to distinguish the relative level of relational versus institutional trust. The questions were as follows: “*You need to rent a house in Yooomhaa, then you will choose: A. houses shown and recommended by agents; B. houses recommended by your friends' friend*”. In this item, institutional trust would be scored one point for choosing option A, and relational trust would be scored one point for choosing option B. The sequence of two kinds of options was ABBA balanced. Finally, the sum of relational and institutional trust was counted separately. Due to the setting of forced opposing options, the correlation between two kinds of trust was −1.00. Cronbach's alpha for these items was .74.

##### Control variables

To control the potential effect of social reality, a variable called social perception (i.e., the subjective perception of soundness of social institutions in general), was measured by three self‐developed items (see Appendices; *α* = .85). It could present individuals' subjective impression on social reality simply and quantifiably, which could be used for eliminating the possible effect of social reality as well as testing whether the setting of fictitious social background was successful. Participants were reminded that “*Now please return to the real world, where you have lived and worked for a long time*” in advance. Then they were asked to evaluate the degree of healthiness or completeness of the overall social institutions on a seven‐point scale, with questions like, “*What do you think of the healthiness of various social institutions today?*” (1 = *totally unhealthy* to 7 = totally *healthy*). When the average score was higher (*M* = 4.18, SD = 1.02), social perception was greater. Demographic variables such as participants' gender, age, level of education, and subjective social status (by a subjective assessment of the 10 rungs ladder; Adler et al., 2000) were recorded (*M* = 4.54, SD = 1.45). In the following analysis, two groups revealed no difference in demographic variables (*p*s > .50), and there were no significant correlations (*p*s > .43) between social perception and other variables, indicating the setting of the fictitious social background was successful to shield the potential confounding effect of social reality.

### Results

**FIGURE 2:**
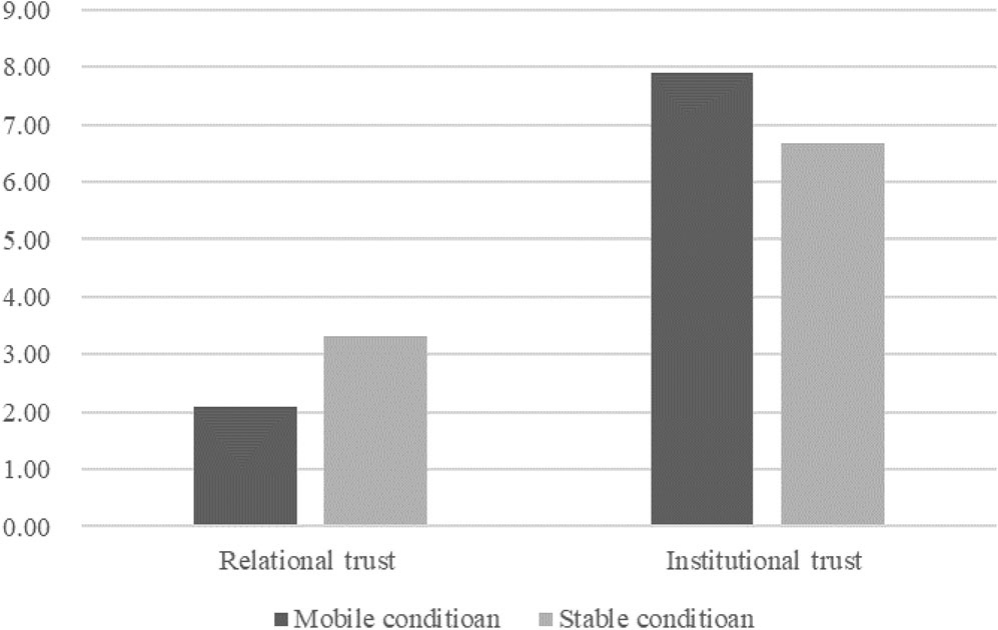
The relationship between residential mobility and two kinds of trust in Study 2.

An independent sample *t*‐test was performed in Study 2, with the priming of residential mobility as a between‐subjects factor. It revealed the score of two kinds of trust was significantly different between two groups (*t*(87) = 2.53, SE = 0.48, *p* = .013, Cohen's *d* = 0.53, 95% CI for relational trust = [0.26, 2.19], 95% CI for institutional trust = [−2.19, −0.26]). The score of relational trust in the mobile condition (*M* = 2.10, SD = 1.95) was significantly lower than that in the stable condition (*M* = 3.33, SD = 2.62), but the score of institutional trust in the mobile condition (*M* = 7.90, SD = 1.95) was significantly higher than that in the stable condition (*M* = 6.68, SD = 2.62). This finding demonstrated that, after being primed with the mindset of residential mobility, participants' relational trust decreased and their institutional trust increased, with a medium effect size (see Figure 2).

### Discussion

Study 2 further examined the causal relationship between residential mobility and two kinds of trust at the individual level. Consistent with the hypotheses, results showed that the priming of residential mobility would significantly lower relational trust and enhance institutional trust. When participants imagined themselves living in a society with high residential mobility (vs. stability), they were more likely to choose to trust institutions over acquaintances among a range of daily life decisions.

Furthermore, Study 2 expands the research paradigm of trust. It created the situational selection task of trust, providing a novel and effective experimental research method for the subsequent research on trust. On the one hand, it fully considers the real situation of Chinese society. Compared to measuring the degree of trust towards objects or rating the degree of agreement in questionnaires, taking choices in specific situations as the representation of individual trust mode will make participants feel that the study is simple, clear and interesting, responding more accurately in the experimental process. On the other hand, the design of forced choices quantifies the trust tendency successfully. It facilitates the collection and analysis of data, and avoids the possible interference of the concept of money in the trust game. In short, by guaranteeing the internal validity of the experimental design, this new research method improved the ecological validity of the experiment by combining the daily life experience of the public. In the process of social change, most previous research on trust used large sample surveys to describe the macro trend, rarely focusing on individuals, which made it difficult to grasp the relationship between variables or explore the change mechanism (Xin, 2019). This new method may provide a good inspiration for the subsequent research on trust in Chinese society.

Lastly, it was noteworthy that the mobile condition and the stable condition both showed relatively high institutional trust. It might be related to the common characteristics of these participants, for young people living in big cities tend to have a high baseline of institutional trust. From the internal aspect, these participants were mostly post‐1990s undergraduates. A survey showed that respondents born in the 1990s had a significantly higher degree of trust in the majority of people in society than those born in earlier generations, and the education level of respondents was proportional to the trust of occupational groups (Gao, 2016b), which could support it indirectly. From the external aspect, they were living in a modern metropolis in China. It is certain that the soundness of institutions in big and modern cities will be far better than in small cities and rural places in China. Therefore, it was understandable that these participants showed relatively high levels of institutional trust, which was similar to the result about the inflow rate and institutional trust in Study 1. Alternatively, even though both groups had high levels of institutional trust, there were also significant differences. After being primed, the already high institutional trust of the mobile group became even higher. The significantly different result between the two groups showed that the effect of residential mobility was real. The prime of residential mobility did enhance institutional trust beyond the characteristics of the participants.

## GENERAL DISCUSSION

In the process of social transformation from traditional society to modern society in China, residential mobility is an important issue. Taking residential mobility as the entry point, the present study attempted to present the psychological transformation of individuals' trust mode caused by social change in two studies.

Study 1 explored the correlational relationship between regional residential mobility and two kinds of trust through large‐scale surveys. It showed that the higher the separation rate, the lower the relational trust (marginally significantly), and the higher the inflow rate, the higher the institutional trust (significantly), consistent with our hypotheses. These results indicated that important changes in interpersonal environment were closely related to people's interaction strategies in interpersonal environment, which reflected indirectly that people would follow changes of social environment and experience corresponding changes in the inner world, that is, the process of adaptation emphasized by socioecological psychology.

Study 2 explored the causal relationship between residential mobility and two kinds of trust using a laboratory experiment. After being primed with the mindset of residential mobility, it showed that institutional trust of the mobile group was significantly higher than that of the stable group, and relational trust of the mobile group was significantly lower than that of the stable group, successfully verifying the hypotheses. Study 2 put forward an empirical research method suitable for the study of Chinese people's trust, and obtained relatively ideal results, which might inspire subsequent researchers to carry out more empirical research on the topic of social change and psychological transformation.

To sum up, these findings confirmed our hypotheses that residential mobility would reduce individuals' relational trust and increase individuals' institutional trust, which meaningfully extended the study of both the psychological effect of residential mobility and the social environmental antecedent of trust.

### Theoretical and practical implications

The present research has much theoretical and practical value. Firstly, the positive effect of residential mobility was highlighted in the present research whereas previous studies have generally focused on its dark side. To some extent, residential mobility is the driver of social trust from traditional relational trust to modern institutional trust. From the perspective of social development, settlements tend to limit people's horizons, foster laziness and dependency, and cause people to yield to customs and traditions to some extent. Conversely, mobility will serve as a new stimulus to people, promote the growth of civilization, and propel the basis of society to transform from kinship relationships to contractual relationships. Thus, the traditional stable society (i.e., “*Gemeinschaft*”) will develop into a modern, changing, law‐based society (i.e., “*Gesellschaft*”). The decline of relational trust and the rise of institutional trust in the mobile environment might be the psychological reflection of this social transformation, in which residential mobility plays a very important role, reflecting the profound impact of residential mobility, a kind of socioecological factor, on people's inner world.

Secondly, trust varies adaptively along the change of social environment, but this process is not easy. Even though our hypotheses were verified successfully, both the mixed results of Study 1 and the fictitious social background in Study 2 implied that the changing process of trust was easily distracted by real social realities. It could be inferred that while relational trust has been decreasing, institutional trust has not been established completely and synchronously in the real world, which might explain the data about the trust crisis in the introduction and reflect the long and tough process of the transformation from relational trust to institutional trust in the real society. Interestingly, the present study also revealed a possible key point of this changing process, that is, the quality of social institutions. It has been discussed above that when social institutions are able to support individuals' reasonable needs in social life (Study 1), and individuals can live in environments with relatively better social institutions (Study 2), institutional trust is high. This implies that the quality of social institutions, that is, the degree of the soundness of social systems in real life, might have an essential influence on institutional trust, and hence the low level of institutional trust might not mean the distrust of social institutions, but the distrust of imperfect social institutions. In some way, institutional trust might be regarded as a sensitive detection testing whether social reality was satisfactory. Therefore, social administrators should pay more attention to taking effective actions to ensure people experience benefits from social institutions and institutional trust, in order to facilitate the changing process of trust among people.

Thirdly, the positive correlation of two kinds of trust provides important information about the inner relationship between them. Study 1 showed the significant positive correlation between relational trust and institutional trust (*r* = .37, *p* < 0.001), which was consistent with results in the previous related studies (e.g., Gao, 2016a). It implied that these two kinds of trust were different but not completely opposite (Liu & Lin, 2013), having some inner link. On the one hand, both of them belong to the mentality of trust, a kind of positive expectation of others' intents and behaviors, and their positive correlation is understandable for they are consistent in the nature of psychological state. On the other hand, under the social situation full of complex problems, institutional trust may not develop suddenly by itself, but has to be generated in virtue of relational trust. It was found in previous research based on CGSS 2005 that Chinese people's trust towards acquaintances and trust towards strangers was not a zero‐sum but positive‐sum relationship (*r* = .337, *p* < .01) and the former was helpful to improve the latter (*B* = 0.353, *p* < .001) (Gao, 2016a). Therefore, it could be induced similarly that relational trust may play an important role in promoting or supplementing institutional trust under the current situation of residential mobility (Gao, 2016a). Additionally, although Study 2 showed completely negative correlation between two kinds of trust due to the unique experimental design, it was not contradictory either, as Study 2 aimed to investigate the specific psychological process and behavioral choices about trust under specific life contexts, whereas Study 1 focused on the overall tendency, which all provided valuable results about two kinds of trust combing different perspectives.

### Limitations and future directions

This study also has some limitations, which might be useful for future research. First, due to the limited access to existing data, only a cross‐sectional analysis was conducted at the regional level. Future studies can attempt to accumulate long‐term data or explore new indicators of residential mobility at the regional level to capture the dynamic characteristics of mobility over time more accurately. In addition, future studies can explore the psychological mechanism, namely, how residential mobility exerts an effect on two kinds of trust, whether through a common cognitive mechanism (e.g., the perception of the instability in interpersonal environment) or different mediating variables (i.e., lowering relational trust by lowering identification of traditional relationalism or enhancing institutional trust by enhancing identification of modern contractarianism). Moreover, this study only divided trust into two static categories, but the inner relationship and dynamic interaction between relational trust and institutional trust could be explored further. Subsequent studies can consider motivational factors of residential mobility, for example, distinguishing between past and expected mobility, investigating separately the active migrants and relocated residents forced by external factors. Other possible positive aspects to residential mobility can also be explored (Choi & Oishi, 2020), such as forming new social norms as well as promoting the development of flexible, creative and diverse perspectives.

## FUNDING INFORMATION

This study was supported by funds from the National Natural Science Foundation of China (Grant No. 31971012).

## CONFLICT OF INTEREST STATEMENT

The authors declare that they have no conflicts of interest.

## ETHICS STATEMENT

All procedures involving human participants in this study were approved by the Ethics Review Committee of the Faculty of Psychology, Beijing Normal University, and in accordance with the 1964 Helsinki Declaration and its later amendments or comparable ethical standards.

## Data Availability

The datasets generated and/or analyzed during the study are available from the corresponding author on reasonable request.

## A. ITEMS IN THE SITUATIONAL SELECTION TASK IN STUDY 2

You are still living in the fictitious society Yooomhaa, where the economy is developed, social institutions are perfect, and daily life is convenient, namely an ideal livable world. How will you choose when you come across the life situations below? Please choose the most preferred action between two options for every item.

1. You want to change your job in Yooomhaa, and then you will choose to:
  - invite friends and family to recommend job opportunities to you.
  - look for opportunities at employment agencies or job boards online.
2. You need to rent a house in Yooomhaa, and then you will choose:
  - houses shown and recommended by agents.
  - houses recommended by your friends' friend.
3. As the project leader of the company, it's time for bidding, and then you will choose to:
  - focus on improving the product, perfecting the bid book and preparing the bid process.
  - contact the leader of the target company for insider information through various connections.
4. You have got married in Yooomahaa and want to buy a car now, and then you will choose to:
  - ask relatives what kinds of cars on the market now are highly cost‐effective.
  - select kinds of cars according to experts' analysis on new products based on various professional indictors by watching the car channel.
5. During holidays and festivals, you want to buy some special products produced by other places, and then you will choose to:
  - order products through mature e‐commerce platforms with service quality supervision.
  - contact relatives and friends who live in those places to help your to inquire, select and purchase products.
6. In Yooomhaa, when you have some contract disputes with other companies in the process of cooperation, you will choose to:
  - try to foster relationship between each other and defuse disagreements with the aid of colleagues who are known well to both sides.
  - resolve the problem through the process of judicial court mediation.
7. When it is necessary to apply for important documents in Yooomhaa's social life (such as birth certificate, various licenses, etc.), you will choose to:
  - get acquaintances to take you to call on the critical person in the application process for the important seal of the documents.
  - go to relevant organizations to deal with these issues, waiting for your turn by number.
8. In Yooomhaa, when you have to select kindergartens or schools for your children, you will choose to:
  - refer to the precise description and recommendation list of schools provided by the education authority after survey and evaluation in the whole district.
  - ask your colleagues who have children and get them to recommend appropriate schools.
9. You feel uncomfortable suddenly and must have an operation as soon as possible after the initial medical examination, and then you will choose to:
  - prepare the red paper containing money as a gift hurriedly and ask acquaintances to find doctors they know well to treat yourself.
  - obtain medical support through the whole procedure in hospital such as register, triage, examination and treatment.
10. You want to know Yooomhaa's social news in leisure time, and then you will choose to:
  - read various official accounts on Weibo or Wechat to learn about events.
  - read messages and comments from friends in group chat.

## B. ITEMS FOR SOCIAL PERCEPTION IN STUDY 2

What do you think of the healthiness of various social institutions nowadays? (1 = totally unhealthy to 7 = totally healthy).

What do you think of the soundness of various social institutions nowadays? (1 = totally unsound to 7 = totally sound).

How satisfied are you with various social institutions nowadays? (1 = totally unsatisfied to 7 = totally satisfied).

## References

[pchj693-bib-0001] Adler, N. E. , Epel, E. S. , Castellazzo, G. , & Ickovics, J. R. (2000). Relationship of subjective and objective social status with psychological and physiological functioning: Preliminary data in healthy white women. Health Psychology, 19(6), 586–592. 10.1037/0278-6133.19.6.586 11129362

[pchj693-bib-0002] Bryk, A. S. , & Raudenbush, S. W. (1992). Hierarchical linear models. Sage Publications.

[pchj693-bib-0003] Cheng, M. Y. , & Duan, C. R. (2021). Highly active population movements in China get further confirmation. Population Research, 45(3), 75–81.

[pchj693-bib-0004] Choi, H. , & Oishi, S. (2020). The psychology of residential mobility: A decade of progress. Current Opinion in Psychology, 32, 72–75. 10.1016/j.copsyc.2019.07.008 31401423

[pchj693-bib-0005] Davis, J. H. , Schoorman, F. D. , Mayer, R. C. , & Tan, H. H. (2000). The trusted general manager and business unit performance: Empirical evidence of a competitive advantage. Strategic Management Journal, 21(5), 563–576. 10.1002/(sici)1097-0266(200005)21:5<563::aid-smj99>3.0.co;2-0

[pchj693-bib-0006] Duan, C. R. , Xie, D. H. , & Lv, L. D. (2019). Migration transition in China. Population Research, 43(2), 12–20.

[pchj693-bib-0007] Faul, F. , Erdfelder, E. , Buchner, A. , & Lang, A. G. (2009). Statistical power analyses using G*power 3.1: Tests for correlation and regression analyses. Behavior Research Methods, 41(4), 1149–1160. 10.3758/brm.41.4.1149 19897823

[pchj693-bib-0008] Fei, X. T. (1985). From the soil: The foundations of Chinese society. SDX Joint Publishing Company.

[pchj693-bib-0009] Gao, X. D. (2016a). Duplication or creation: Reconstructing path of interpersonal trust based on perspective of social mobility. Journal of Social Sciences, 06, 71–83. 10.13644/j.cnki.cn31-1112.2016.06.007

[pchj693-bib-0010] Gao, X. D. (2016b). Report on social trust in China (2016). In J. X. Wang & M. Q. Chen (Eds.), Annual report of social mentality of China (2016) (pp. 117–141). Social Sciences Academy Press.

[pchj693-bib-0011] Hafeez, A. , Dangel, W. J. , Ostroff, S. M. , Kiani, A. , Glenn, S. , Abbas, J. , Afzal, M. S. , Afzal, S. , Ahmad, S. , Ahmed, A. S. , Ahmed, H. , Ali, L. , Ali, M. , Ali, Z. , Arshad, M. , Ashraf, T. , Bhutta, Z. A. , Bibi, S. , Butt, Z. A. , … Mokdad, A. H. (2023). The state of health in Pakistan and its provinces and territories, 1990–2019: A systematic analysis for the Global Burden Of Disease Study 2019. The Lancet. Global Health, 11, e229–e243. 10.1016/s2214-109x(22)00497-1 36669807 PMC10009760

[pchj693-bib-0012] Hu, R. , & Li, J. Y. (2006). The composition of urban residents' trust and its influencing factors. Chinese Journal of Sociology, 26(6), 45–61. 10.15992/j.cnki.31-1123/c.2006.06.006

[pchj693-bib-0013] Jing, S. J. (2013). Conceptualizing and measuring sense of social trust. In Y. Y. Yang & J. X. Wang (Eds.), Research on social mentality in contemporary China (pp. 108–135). Social Sciences Academic Press.

[pchj693-bib-0014] Knack, S. , & Keefer, P. (1997). Does social capital have an economic payoff? A cross‐country investigation. Quarterly Journal of Economics, 112(4), 1251–1288. 10.1162/003355300555475

[pchj693-bib-0015] Kreft, I. , & De Leeuw, J. (1998). Introducing multilevel modeling. Sage. 10.4135/9781849209366

[pchj693-bib-0016] Li, W. , Li, L. M. , & Li, M. (2019). Residential mobility reduces ingroup favoritism in prosocial behavior. Asian Journal of Social Psychology, 22, 3–17. 10.1111/ajsp.12338

[pchj693-bib-0017] Li, W. M. , & Liang, Y. C. (2002). Particular trust and universal trust: The structure and characteristics of Chinese trust. Sociological Studies, 3, 11–22.

[pchj693-bib-0018] Liu, G. F. , & Lin, C. D. (2013). Constructing trust index and building harmonious society. Journal of Beijing Normal University (Social Sciences), 1, 25–32.

[pchj693-bib-0019] Lun, J. , Oishi, S. , & Tenney, E. R. (2012). Residential mobility moderates preferences for egalitarian versus loyal helpers. Journal of Experimental Social Psychology, 48(1), 291–297. 10.1016/j.jesp.2011.09.002

[pchj693-bib-0020] Lun, J. , Roth, D. , Oishi, S. , & Kesebir, S. (2012). Residential mobility, social support concerns, and friendship strategy. Social Psychological and Personality Science, 4(3), 332–339. 10.1177/1948550612453345

[pchj693-bib-0021] McNamara, R. A. , & Henrich, J. (2017). Kin and kinship psychology both influence cooperative coordination in Yasawa, Fiji. Evolution and Human Behavior, 38, 197–207. 10.1016/j.evolhumbehav.2016.09.004

[pchj693-bib-0022] Oishi, S. (2010). The psychology of residential mobility: Implications for the self, social relationships, and well‐being. Perspectives on Psychological Science, 5(1), 5–21. 10.1177/1745691609356781 26162059

[pchj693-bib-0023] Oishi, S. (2014). Socioecological psychology. Annual Review of Psychology, 65, 581–609. 10.1146/annurev-psych-030413-152156 23987114

[pchj693-bib-0024] Oishi, S. , Ishii, K. , & Lun, J. (2009). Residential mobility and conditionality of group identification. Journal of Experimental Social Psychology, 45(4), 913–919. 10.1016/j.jesp.2009.04.028

[pchj693-bib-0025] Oishi, S. , & Kesebir, S. (2012). Optimal social‐networking strategy is a function of socioeconomic conditions. Psychological Science, 23(12), 1542–1548. 10.1177/0956797612446708 23129061

[pchj693-bib-0026] Oishi, S. , Kesebir, S. , Miao, F. F. , Talhelm, T. , Endo, Y. , Uchida, Y. , Shibanai, Y. , & Norasakkunkit, V. (2013). Residential mobility increases motivation to expand social network: But why? Journal of Experimental Social Psychology, 49(2), 217–223. 10.1016/j.jesp.2012.10.008

[pchj693-bib-0027] Oishi, S. , Miao, F. F. , Koo, M. , Kisling, J. , & Ratliff, K. A. (2012). Residential mobility breeds familiarity‐seeking. Journal of Personality and Social Psychology, 102(1), 149–162. 10.1037/a0024949 21843015

[pchj693-bib-0028] Oishi, S. , & Talhelm, T. (2012). Residential mobility: What psychological research reveals. Current Directions in Psychological Science, 21(6), 425–430. 10.1177/0963721412460675

[pchj693-bib-0029] Paulson, K. R. , Kamath, A. M. , Alam, T. , Bienhoff, K. , Abady, G. G. , Abbas, J. , Abbasi‐Kangevari, M. , Abbastabar, H. , Abd‐Allah, F. , Abd‐Elsalam, S. M. , Abdoli, A. , Abedi, A. , Abolhassani, H. , Abreu, L. G. , Abu‐Gharbieh, E. , Abu‐Rmeileh, N. M. , Abushouk, A. I. , Adamu, A. L. , Adebayo, O. , … Kassebaum, N. J. (2021). Global, regional, and national progress towards Sustainable Development Goal 3.2 for neonatal and child health: All‐cause and cause‐specific mortality findings from the Global Burden Of Disease Study 2019. Lancet, 398, 870–905. 10.1016/S0140-6736(21)01207-1 34416195 PMC8429803

[pchj693-bib-0030] Rand, D. G. , & Nowak, M. A. (2013). Human cooperation. Trends in Cognitive Sciences, 17, 413–425. 10.1016/j.tics.2013.06.003 23856025

[pchj693-bib-0031] Rao, Y. S. , Zhou, J. , Tian, Z. B. , & Yang, Y. Y. (2013). A survey on social trust status among urban residents. In Y. Y. Yang & J. X. Wang (Eds.), Annual report of social mentality of China (2012–2013) (pp. 71–93). Social Sciences Academy Press.

[pchj693-bib-0032] Rousseau, D. M. , Sitkin, S. , Burt, R. S. , & Camerer, C. F. (1998). Not so different after all: A cross‐discipline view of trust. Academy of Management Review, 23(3), 393–404. 10.5465/amr.1998.926617

[pchj693-bib-0033] Sánchez‐Rodríguez, Á. , Willis, G. B. , & Rodríguez‐Bailón, R. (2017). Economic and social distance: Perceived income inequality negatively predicts an interdependent self‐construal. International Journal of Psychology, 54(1), 117–125. 10.1002/ijop.12437 28675432

[pchj693-bib-0034] Schug, J. , Yuki, M. , & Maddux, W. (2010). Relational mobility explains between‐ and within‐culture differences in self‐disclosure to close friends. Psychological Science, 21(10), 1471–1478. 10.1177/0956797610382786 20817913

[pchj693-bib-0035] Tao, Z. L. , & Wang, H. (2006). The change of trust mode: From interpersonal trust to institution‐based trust. Journal of Beijing University of Posts and Telecommunications (Social Sciences Edition), 8(2), 20–23.

[pchj693-bib-0036] Tong, Z. F. (2006). Chaxu Geju of trust: An explanation of interpersonal trust in rural society. Gansu Theory Research, 3, 59–63.

[pchj693-bib-0037] Wang, Y. W. , Chen, L. C. , & Hwang, K. K. (2006). The strategies of trust in Chinese society. Indigenous Psychology Research, 25, 199–242.

[pchj693-bib-0038] Xin, Z. Q. (2019). Marketization and interpersonal trust decline in China. Advances in Psychological Science, 27(12), 1951–1966. 10.3724/sp.j.1042.2019.01951

[pchj693-bib-0039] Zhang, Y. , & Xin, Z. Q. (2019). Rule comes first: The influences of market attributes on interpersonal trust in the marketization process. Journal of Social Issues, 75(3), 1–29. 10.1111/josi.12306

[pchj693-bib-0040] Zhao, N. , Xu, K. Q. , & Sun, L. (2021). Residential mobility and trust: The moderating role of cognitive need for closure. Journal of Pacific Rim Psychology, 15(4), 183449092097475. 10.1177/1834490920974759

[pchj693-bib-0041] Zhou, H. (2021). The stability of migration pattern in China and related issues: Consideration based on the data of the seventh National Census Bulletin. Chinese Journal of Population Science, 3, 28–41.

[pchj693-bib-0042] Zhu, H. (2011). From “trust for affinity” to “trust for interrelated interests”: The shift of interpersonal trust—An empirical study on interpersonal trust. Academia Bimestris, 4, 115–121. 10.13713/j.cnki.cssci.2011.10.026

[pchj693-bib-0043] Zuo, S. J. , Cai, P. , Huang, N. W. , Wang, F. , & Wang, P. (2022). Population migration damages the natural environment: A multilevel investigation of the relationship between residential mobility and pro‐environmental behaviors. Personality and Social Psychology Bulletin, 49(5), 758–772. 10.1177/01461672221079451 35236177

[pchj693-bib-0044] Zuo, S. J. , Huang, N. W. , Cai, P. , & Wang, F. (2018). The lure of antagonistic social strategy in unstable socioecological environment: Residential mobility facilitates individuals' antisocial behavior. Evolution and Human Behavior, 39(3), 364–371. 10.1016/j.evolhumbehav.2018.03.002

